# A novel approach to estimating $R_{t}$ through infection networks: understanding regional transmission dynamics of COVID-19

**DOI:** 10.3389/fpubh.2025.1586786

**Published:** 2025-06-18

**Authors:** Byul Nim Kim, Junwoo Jo, Chunyoung Oh, Sanghyeok Moon, Arsen Abdulali, Sunmi Lee

**Affiliations:** ^1^Department of Applied Mathematics, Kyung Hee University, Yongin, Republic of Korea; ^2^Department of Mathematics Education, Chonnam National University, Gwangju, Republic of Korea; ^3^Department of Engineering, University of Cambridge, Cambridge, United Kingdom

**Keywords:** empirical effective reproduction number, infection network, COVID-19, South Korea, region-specific transmission

## Abstract

**Introduction:**

The effective reproduction number ($R_{t}$) is a key indicator for monitoring and controlling infectious diseases such as COVID-19, where transmission patterns can differ substantially across demographics, regions, and phases of the pandemic. In this study, we propose a novel, network-based approach to empirically estimate $R_{t}$ using detailed transmission data from South Korea. By reconstructing infector–infectee pairs, our method incorporates local factors like mobility and social distancing, offering a more precise perspective than traditional methods.

**Methods:**

We acquired infector–infectee pair data from the Korea Disease Control and Prevention Agency (KDCA) for 2020–2021 and built infection networks to derive empirical $R_{t}$. This framework allows us to examine regional differences and the effects of social distancing measures. We also compared our results with Cori's $R_{t}$, which employs incidence data and serial interval distributions, to highlight the advantages of an infection network-based strategy.

**Results:**

Our empirical $R_{t}$ uncovered three distinct patterns. Early in the outbreak, when case numbers were low, $R_{t}$ remained near 1, indicating limited transmission. During superspreading events, our estimates showed sharper peaks than Cori's method, demonstrating higher sensitivity to sudden changes. As the Delta variant emerged, our $R_{t}$ values converged with Cori's, underscoring the utility of network-based methods for capturing nuanced shifts during high-variability phases.

**Discussion:**

Incorporating infection networks into $R_{t}$ estimation thus provides decision-makers with timely insights for targeted interventions. Empirically reconstructing infection networks and directly estimating $R_{t}$ reveal real-time transmission dynamics often overlooked by aggregated approaches. This method can significantly improve outbreak forecasts, inform more precise public health policies, and strengthen pandemic preparedness.

## 1 Introduction

COVID-19 has emerged as one of the most significant global health crises, prompting extensive research aimed at understanding and controlling the spread of the virus.^1^ Effective prediction and management of disease transmission are critical for implementing timely public health interventions. To address this need, numerous studies have investigated the clinical and epidemiological characteristics of COVID-19 (1, 2). A key metric for assessing a virus's contagiousness is the effective reproduction number ($R_{t}$), which represents the average number of secondary infections caused by an infected individual at any given time. This measure is essential for detecting shifts in transmission dynamics, predicting disease spread, and guiding interventions such as social distancing and lockdowns.

Estimating $R_{t}$ presents significant challenges, including incomplete data collection, asymptomatic transmission, and the inherent complexity of disease spread. Traditionally, $R_{t}$ has been estimated through indirect methods that rely on specific assumptions and epidemiological modeling. Commonly used techniques include the Cori's method (often implemented via the EpiEstim R package), which combines case incidence data with a serial interval distribution (3). Other approaches include the Serial Interval Method (3–6), which uses the timing of symptom onset between infector–infectee pairs; the Exponential Growth Method (7), typically applied during the initial rapid spread; the Generation Interval Method (8), derived from contact tracing data; and Time-dependent Methods (9), which use sliding windows for real-time estimates. More advanced frameworks have also emerged. Bayesian Methods incorporate prior knowledge to provide probabilistic estimates (10–13), while Agent-based and Stochastic Models simulate individual-level transmission (14, 15). Kalman Filtering can update $R_{t}$ in real time as data accrue (16, 17), and Regression Techniques help quantify the relationship between $R_{t}$ and specific public health interventions (18). Although each of these methods provides unique insights based on the outbreak phase and available data, they are often indirect and rely on simplifying assumptions. These assumptions can introduce discrepancies between model predictions and actual transmission patterns, highlighting the need for more direct approaches.

Infection networks offer a complementary, data-driven method for understanding how diseases like COVID-19 propagate. Individuals are represented as nodes, and edges denote transmission events. By tracing these links, infection networks reveal the routes through which a virus spreads in a population, enabling precise identification of transmission chains and clusters. Temporal contact networks—those accounting for the timing of interactions—further enhance our understanding of disease spread compared to static models (19). A significant advantage of infection networks lies in the direct calculation of the empirical $R_{t}$. Rather than relying on indirect estimations, one can measure how many individuals each infected person actually infects, in real time, thereby improving the accuracy and timeliness of $R_{t}$ estimates for intervention evaluations and outbreak forecasting. South Korea provides a particularly informative case study for applying this network-based approach. Its government implemented rigorous contact tracing early in the pandemic, gathering detailed transmission data. Although explosive outbreaks eventually strained these tracing efforts, South Korea's extensive records—curated by the Korea Disease Control and Prevention Agency (KDCA)—include detailed information on symptom onset, diagnosis, transmission routes, and demographic factors such as age and region (20, 21). Despite partial data gaps, this high-resolution dataset offers a unique opportunity to study COVID-19 dynamics via empirical infection networks.

In addition to these data resources, a variety of mathematical models incorporating age structure or spatial heterogeneity (22, 23), as well as network-based frameworks (24, 25), have been developed to capture the nuanced dynamics of infectious diseases. While these models are grounded in theoretical formulations, they differ from our empirical approach. Both perspectives, however, underscore the significant impact of population heterogeneity on disease transmission. Numerous methods exist for estimating $R_{t}$, including the exponential growth method, the Wallinga–Teunis approach, and Cori's method. Although each provides valuable epidemiological insights, they often rely on aggregated data and assume fixed serial intervals under homogeneous mixing, limiting their capacity to capture variation across distinct demographic groups, regions, and time frames. By contrast, South Korea's contact tracing data allow us to reconstruct infection networks and calculate $R_{t}$ directly from observed infector–infectee relationships. To address potential gaps in this dataset, we apply exponential degree modeling and bootstrap sampling techniques to handle incomplete contact information more effectively.

In this study, we introduce a novel network-based approach for estimating the empirical $R_{t}$ using detailed COVID-19 transmission data from South Korea. By constructing infection networks grounded in infector–infectee pairs, our method captures key real-world features—such as outbreak timing and superspreading events—that are often overlooked in more traditional, model-based frameworks. This empirical approach yields a more context-sensitive measure of transmission, especially in heterogeneous settings. By accounting for factors like age, regional distinctions, mobility trends, and social distancing measures, our method provides deeper insights into the virus's spread and evolution. Ultimately, this research is significant because it advances our capacity to capture and understand the dynamics of infectious diseases in a manner that more closely reflects real-world conditions. Our network-based approach can inform evidence-based interventions and enhance epidemic forecasting, thus supporting more effective and timely public health strategies in current and future pandemics.

## 2 Materials and methods

### 2.1 COVID-19 infection network

We utilize COVID-19 data obtained from the Korea Disease Control and Prevention Agency (KDCA), covering the period from February 1, 2020, to December 31, 2021, during which a total of 670,484 confirmed cases were reported.^2^ A key aspect of this dataset is its detailed recording of both infectors and infectees, allowing for the construction of an extensive infection network. This structured network provides a crucial foundation for developing novel approaches to computing the effective reproduction number, which is essential for understanding transmission dynamics and evaluating intervention strategies. Epidemiological teams collected comprehensive information on infectors and infectees, including demographics, symptom onset, diagnosis, and age. Contact tracing was systematically performed using the COVID-19 Epidemiological Investigation Support System (K-EISS), enabling the reconstruction of transmission pathways with high accuracy (26). Regional COVID-19 analysis teams ensured precise validation of the collected data, further strengthening the reliability of the infection network.

**Figure 1:**
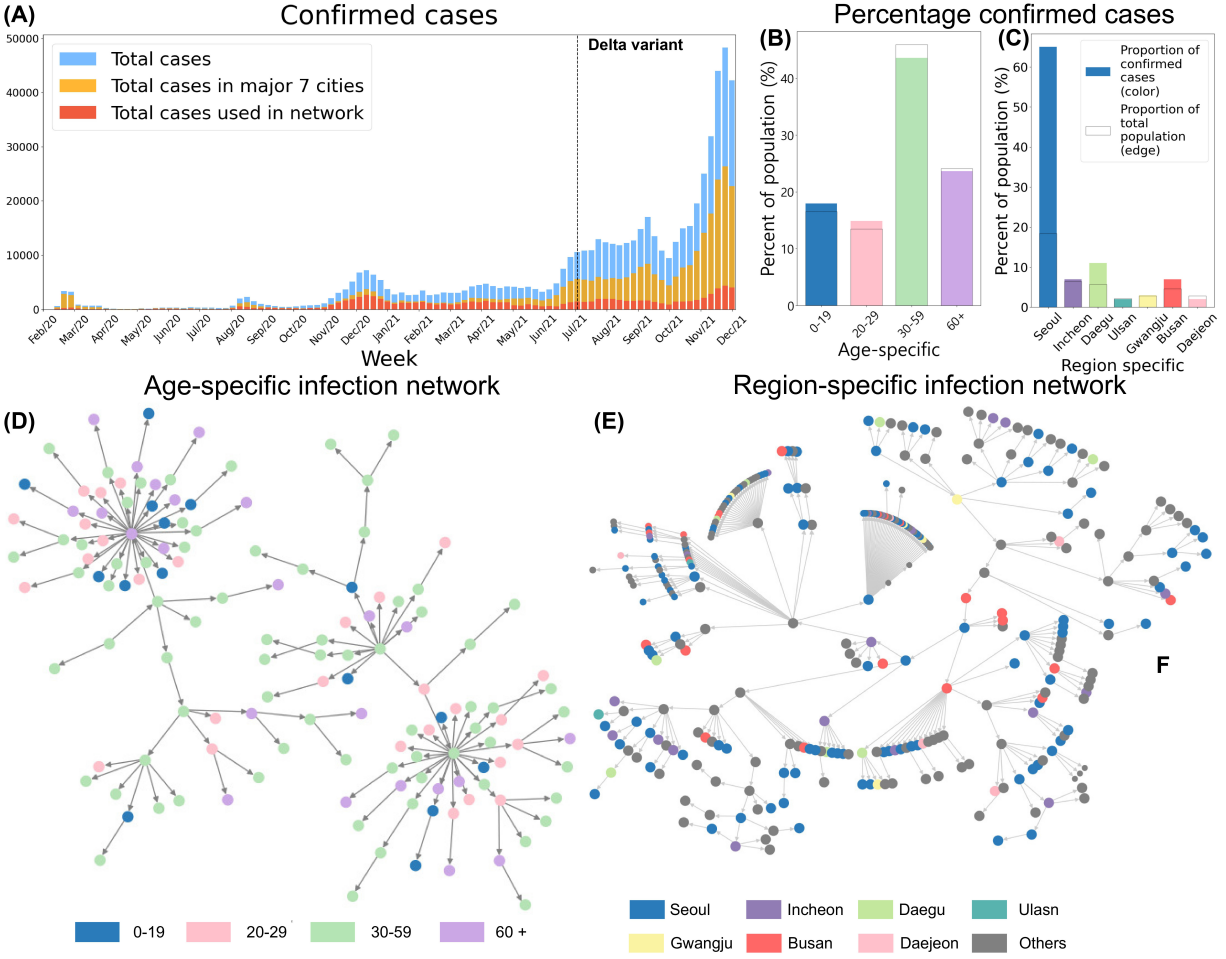
**(A)** Total confirmed cases, all cases in major 7 regions and the cases in 7 region-specific networks are shown. **(B)** Proportion of confirmed cases by age group. **(C)** Proportion of confirmed cases by region. **(D)** Age-specific Infection Network. **(E)** Region-specific Infection Network, and nodes colored based on regions.

We constructed infection networks by stratifying infected individuals into four age groups (0–19, 20–29, 30–59, and 60+) and by distinguishing between metropolitan (Seoul, Incheon) and non-metropolitan cities (Daegu, Ulsan, Gwangju, Busan, Daejeon). These networks enabled us to trace transmission pathways and create directed infection trees, where each node corresponds to an infected individual and each edge indicates a transmission link from an infector to an infectee. Moreover, this approach allows us to reconstruct and visualize the observed transmission trajectories, rather than relying on predefined or synthetic network configurations. Figure 1 provides an overview of confirmed cases by age and region, as well as the infection networks used to compute the empirical $R_{t}$. In Figure 1A, blue bars represent the total number of confirmed cases, yellow bars indicate cases in the seven major cities, and red bars denote cases with complete contact-tracing data (used to build the infection network). Figures 1B, C depict the proportion of confirmed cases by age group and region, respectively. The outlined bars show each group's share of the total population, whereas the colored bars represent the actual proportion of confirmed cases. Figures 1D, E illustrate the resulting infection networks, stratified by age group and region. Before the Delta variant became dominant, 55% of all nodes were part of connected components spanning all regions. After the Delta variant's emergence, this proportion declined to 28%. Detailed statistics on nodes and edges within region-specific and age-specific networks can be found in Tables 1, 2.

**Table 1:** The age-specific network includes nodes representing confirmed cases, with linked nodes identified through contact tracing and unlinked nodes lacking such connections.

**Age**	**Nodes**	**Linked nodes**	**Unlinked nodes**	**Edges**
0–19	113,558	57,774	55,784	39,644
20–29	93,937	35,146	58,791	22,167
30–59	275,730	110,982	164,748	74,383
60+	149,577	62,369	87,208	43,067

Edges represent transmission chains, categorized across four distinct age groups.

**Table 2:** The region-specific network consists of nodes representing confirmed cases, with linked nodes identified through contact tracing and unlinked nodes lacking such connections.

**Region**	**Nodes**	**Linked nodes**	**Unlinked nodes**	**Edges**
Seoul	219,895	37,350	182,545	27,230
Incheon	37,949	8,082	29,867	6,162
Daegu	22,945	14,508	8,437	12,106
Ulsan	6,885	5,201	1,684	4,127
Gwangju	8,097	6,812	1,285	5,638
Busan	25,313	19,163	6,150	15,287
Daejeon	12,435	8,984	3,451	7,053

Edges denote transmission chains, categorized into seven major regional groups.

### 2.2 Empirical effective reproduction number

In our infection networks, each node represents an individual, and each directed edge denotes the transmission link between an infector and an infectee. Infector nodes have outgoing edges, indicating the spread of infection to others, while infectee nodes have incoming edges, representing transmission from a source. Each node is associated with relevant attributes, including report date, age, and residence area, allowing for a detailed reconstruction of transmission pathways. To quantify transmission dynamics, we calculate the empirical reproduction number $R_{t}$, which reflects the average number of secondary infections generated by an infector at time *t*. Unlike theoretical estimates derived from compartmental models, our empirical $R_{t}$ is directly computed from the infection network by averaging the number of infectees linked to each infector in the infection tree. This approach provides a data-driven measure of disease spread, capturing real-world transmission patterns and temporal variations in infectiousness. By leveraging network-based calculations, our method offers a more precise representation of outbreak dynamics, enabling a deeper understanding of how infections propagate across different demographic and geographic groups. Furthermore, this approach enables us to capture local and temporal fluctuations such as superspreading events.

To quantify transmission dynamics from our infection network data, we define the empirical effective reproduction number $R_{t}$ as the average number of secondary infections generated per infector within a rolling *n*-day window. Each time point *t* corresponds to the end of a 7-day period (week *t*), with the calculation incorporating data from the preceding *n* days, ending on day 7*t*. The parameter *n* controls the length of this rolling window, thereby balancing temporal resolution and smoothness in the $R_{t}$ estimate. While a smaller *n* (e.g., 3–5 days) can capture rapid fluctuations more effectively but may introduce noise, a larger *n* (e.g., 10–14 days) produces smoother estimates at the cost of delayed responsiveness. Here, we set *n* = 7 to align with both weekly public health reporting cycles and the need for a stable yet responsive measure of epidemic dynamics. Specifically, we construct a sequence of daily infection networks *G*_*k*_ = (*V*_*k*_, *E*_*k*_), where:

- *V*_*k*_ denotes the set of individuals infected on day *k*,
- *E*_*k*_ ⊆ *V*_*k*_ × *V*_*k*_ is the set of directed edges representing transmission links, where each edge (*i, j*) ∈ *E*_*k*_ indicates that individual *i* transmitted the infection to individual *j* on day *k*.

Let $V_{k}^{\prime }\subseteq V_{k}$ denote the set of infectors on day *k*, defined as those nodes with at least one outgoing edge (i.e., out-degree ≥1). We then define the empirical effective reproduction number $R_{t}$ as:


(1) $$\begin{array}{l} R_{t}=\frac{\overset{7t}{\underset{k=\max\left(1,7t-n+1\right)}{\sum }}|E_{k}|}{\overset{7t}{\underset{k=\max\left(1,7t-n+1\right)}{\sum }}|V_{k}^{\prime }|} \end{array}$$

Here, |*E*_*k*_| denotes the number of secondary transmission events (edges) observed on day *k*, and $\left|V_{k}^{\prime }\right|$ is the number of unique infectors on that day. The term max(1, 7*t* − *n* + 1) prevents the summation from exceeding the dataset's range, which is particularly important during the outbreak's initial stages. This empirical approach offers a real-time assessment of disease spread based on observed transmission events, providing a more precise depiction of outbreak dynamics and capturing the heterogeneous nature of COVID-19 transmission.

Nevertheless, this method can overestimate $R_{t}$ when contact tracing capacity is limited, resulting in incomplete networks. To mitigate this issue, we adjusted the denominator by incorporating the proportion of disconnected nodes, thereby reducing the inflation of $R_{t}$ estimates.


(2) $$\begin{array}{l} R_{t}=\frac{\overset{7t}{\underset{k=\max\left(1,7t-n+1\right)}{\sum }}|E_{k}|}{\overset{7t}{\underset{k=\max\left(1,7t-n+1\right)}{\sum }}|V_{k}^{\prime }|+\overset{7t}{\underset{k=\max\left(1,7t-n+1\right)}{\sum }}\alpha \left(\left|V_{k}|-|V_{k}^{\prime }\right|\right)}. \end{array}$$

**Figure 2:**
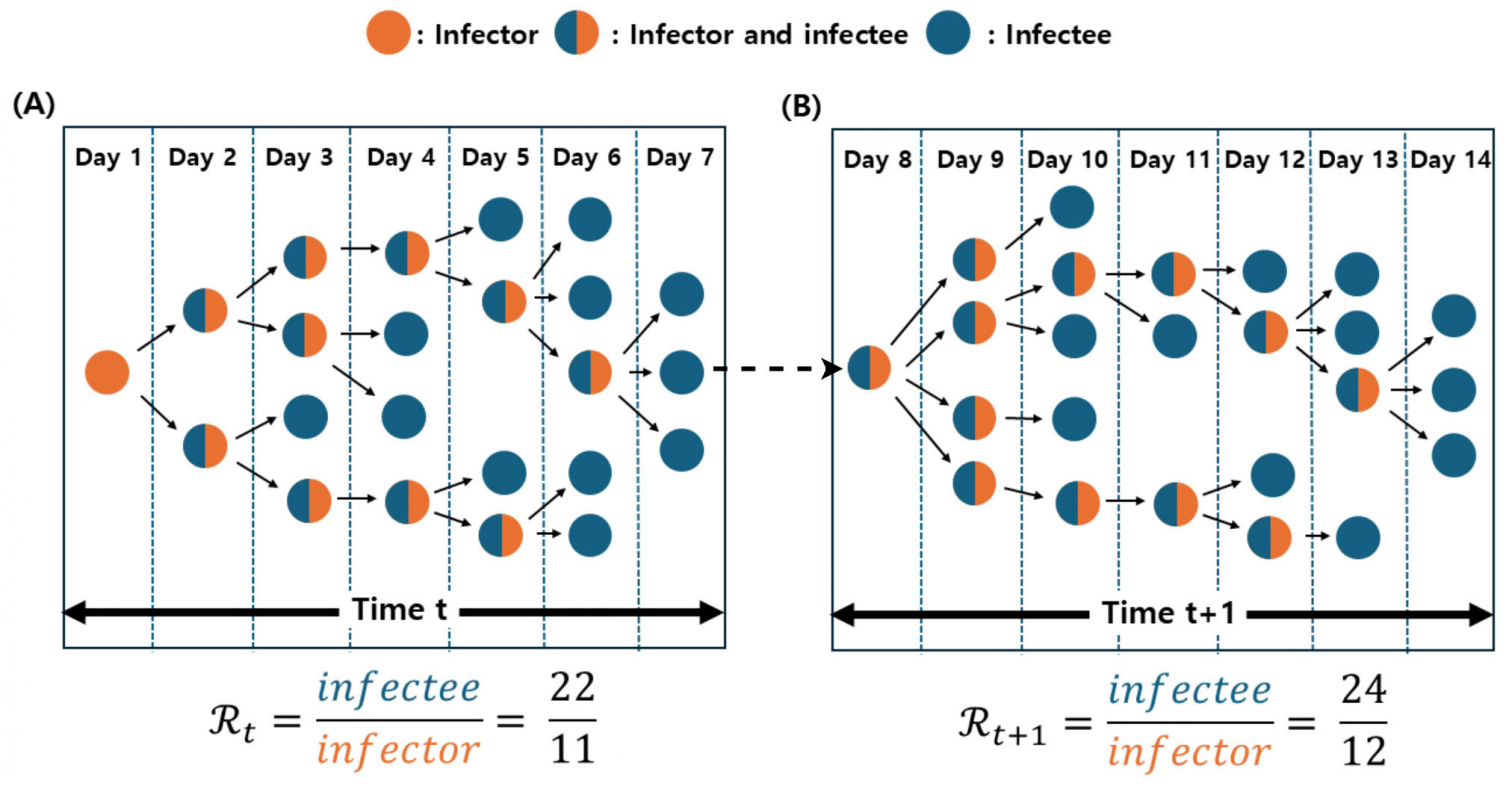
Illustration of the empirical $R_{t}$ calculation at time *t* and *t* + 1. Orange nodes represent infectors, blue nodes indicate infectees, and merged blue-orange nodes denote individuals acting as both infectors and infectees. **(A)** At time *t*, the empirical $R_{t}$ is computed as the ratio of infectees to infectors, yielding a value of 2. **(B)** At time *t* + 1, the network consists of 24 infectees (blue nodes) and 12 infectors (orange nodes), resulting in an empirical $R_{t}$ of 2.

We introduce the parameter α to adjust for incomplete contact tracing, thereby preventing overestimation of the empirical $R_{t}$. In essence, α accounts for potentially underobserved transmission by incorporating a fraction of disconnected nodes into the denominator, approximating a fully connected network under real-world limitations. We included Figure 2 for enhancing the better understanding of calculating empirical $R_{t}$.

We additionally estimated the effective reproduction number using Cori's method (3), a widely applied approach for time-varying $R_{t}$ based on incidence data. In this framework, $R_{t}$ is calculated as the ratio of newly observed cases at time *t* to the total infectiousness of preceding cases, where infectiousness is determined by summing confirmed cases weighted by a serial interval distribution. To maintain consistency with our empirical network-based approach, we used a seven-day sliding window and adopted age- and region-specific serial intervals (4). While Cori's method has been successfully employed in numerous studies (6, 27, 28), its assumption of homogeneous mixing and reliance on aggregated incidence data may overlook complex structural and temporal heterogeneity in transmission dynamics. By contrast, our proposed method reconstructs empirical infection networks from infector–infectee pairs, directly computing $R_{t}$ from observed transmission events. This network-based framework enables stratification by age and region, integrates mobility and policy data, and offers a finer-grained representation of local transmission patterns. As a result, it can capture rapid changes and superspreading events more effectively than methods that assume uniform mixing, particularly in heterogeneous settings where COVID-19 transmission exhibits substantial variability across populations and time.

Contact tracing may fail to capture all transmission links due to factors such as asymptomatic cases, surges in incidence, and inaccuracies in survey responses (29, 30). Nevertheless, our empirical approach relies on constructing a complete infection network. To address potential data incompleteness, we use confidence intervals and estimate unreported transmissions by sampling from a fitted degree distribution. The construction of the confidence interval for the empirical reproduction number ($R_{t}$) assumes that the infection network mirrors the underlying social contact network, highlighting the importance of real-world network structures. In light of data limitations and the complexity of social networks, we employ an exponential degree distribution, which effectively captures heterogeneous contact patterns and is appropriate for incomplete datasets (31). To compute the confidence interval, we repeatedly sample node degrees within the exponential network over a specified time window. From these sampled data, we generate empirical reproduction numbers and define the confidence interval for ($R_{t}$) by selecting the 5%–95% values observed across all samples. The exponential model is advantageous for modeling heterogeneous contact behavior—especially in the presence of partial data—while remaining parsimonious enough to accommodate the long-tailed nature of real-world transmission (31). Our initial analyses revealed right-skewed degree distributions that fit well with an exponential function, allowing for simpler calculations of network metrics and estimates. However, we recognize that true contact networks may be more complex—particularly when superspreader events lead to heavy-tailed degree distributions—and thus plan to evaluate alternative models (e.g., negative binomial or power-law) in future work to further test model robustness and improve realism.

### 2.3 Social distancing measures and mobility

**Figure 3:**
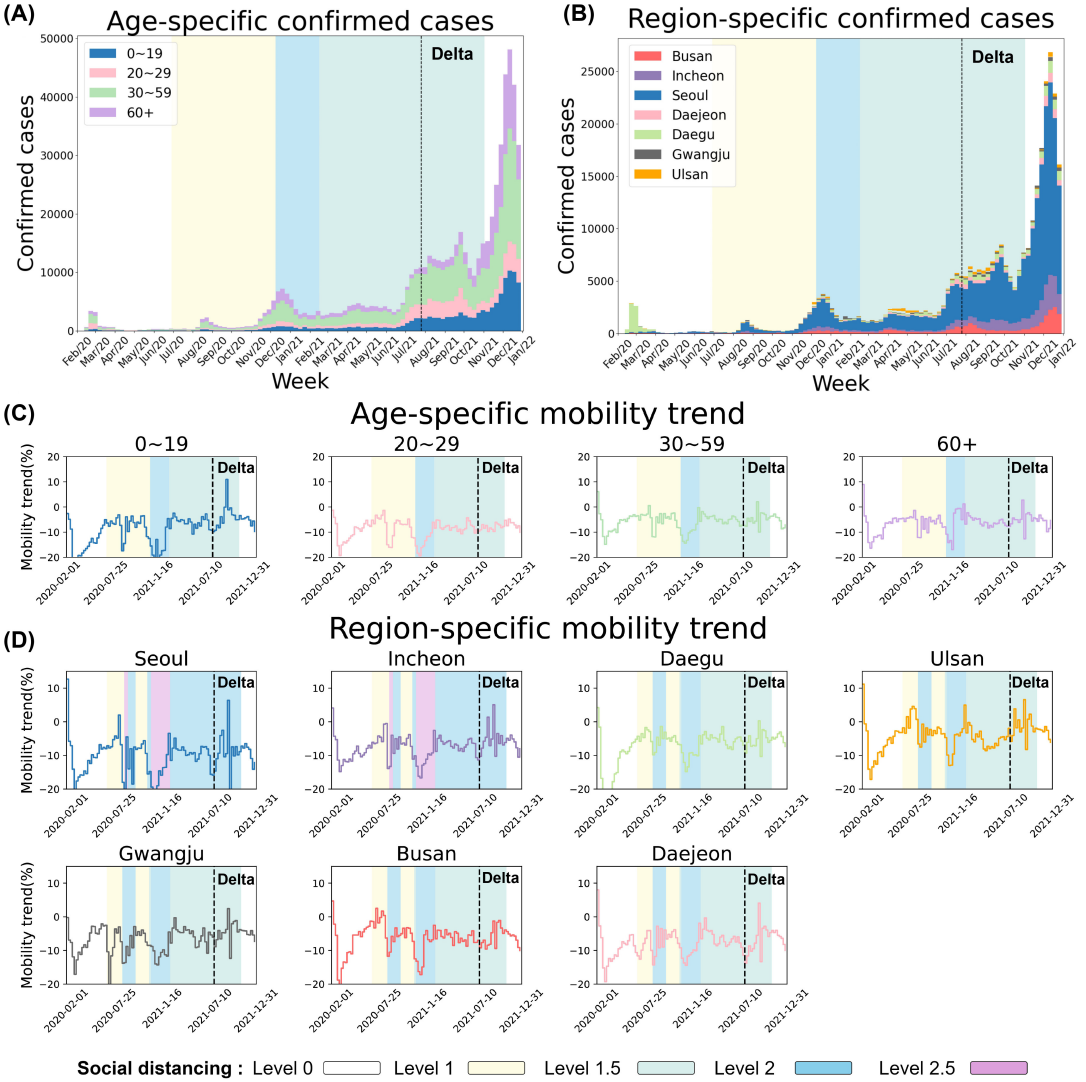
Temporal trends of COVID-19 incidence and mobility by age group and region (2020–2021) compared to 2019. **(A, B)** present the weekly number of confirmed COVID-19 cases across different age groups and regions, highlighting a sharp increase in cases during the dominance of the Delta variant. The black dashed line marks the point at which the Delta variant accounted for more than 50% of cases. **(C, D)** illustrate weekly mobility trends by age group and region, showing changes in mobility relative to 2019 levels. Mobility decreased during periods of stricter social distancing and increased as restrictions were eased. The background shading represents the levels of social distancing policies implemented in non-metropolitan areas over time.

Using COVID-19 confirmed case data, we constructed infection trees for four age groups (0–19, 20–29, 30–59, and 60+) and seven regions (Seoul, Incheon, Daegu, Ulsan, Gwangju, Busan, and Daejeon). We also analyzed confirmed cases and weekly mobility trend from SKT movement data by age and region from February 2020 to December 2021 (32). The background color in Figure 3 shows social distancing levels in non-metropolitan areas, while the black dashed line indicates when the Delta variant exceeded 50% (1). Figures 3A, B show a sharp rise in confirmed cases during the Delta variant's dominance. Figures 3C, D depict weekly mobility trends, which decreased with stricter social distancing and increased as restrictions were relaxed. After the school closure policy was relaxed in August 2021, in-person classes resumed, especially in non-metropolitan areas, leading to increased mobility among younger populations. During the early stage of COVID-19, the initial outbreak led to a noticeable decline in mobility, particularly in Daegu compared to other regions. Despite this decrease, Daegu experienced a rapid surge in confirmed cases. During this period, the mobility rate dropped to approximately 0.7, suggesting that the superspreading event at the church (Table 3) played a pivotal role in driving both the sharp rise in infections and fluctuations in mobility. In contrast, during the Delta variant wave, mobility trends exhibited an overall increase across seven regions, even amid a significant rise in cases. This indicates that, unlike the early outbreak phase, mobility patterns were less influenced by case numbers, likely due to shifts in social distancing policies (33).

**Table 3:** Social distancing levels in metropolitan and non-metropolitan areas (45, 46).

**Timeline**	**Metropolitan area**	**Non-metropolitan area**
2020-06-28	Level 1	Level 1
2020-08-23		Level 2
2020-08-30	Strengthened level 2	
2020-09-14	Level 2	
2020-10-12	Level 1	Level 1
2020-11-24	Level 2	
2020-12-01		Level 1.5
2020-12-08	Level 2.5	Level 2
2021-02-15	Level 2	Level 1.5
2021-11-01	Level 0	Level 0

Social distancing is a public health measure aimed at preventing the spread of infectious diseases by encouraging individuals to maintain physical distance from one another. This policy is designed to block or reduce transmission pathways, thereby slowing the spread of infection. During the COVID-19 pandemic, social distancing was one of the critical disease control strategies implemented globally. In South Korea, social distancing measures were applied at various levels throughout pandemic, depending on the outbreak's severity. These levels were adjusted based on the number of confirmed cases in each region and the strain on the healthcare system. South Korea's social distancing measures were structured into five levels, each progressively strengthened or relaxed according to the situation. The levels of social distancing for metropolitan and non-metropolitan cities over different periods are summarized in Table 4.

**Table 4:** The school closure policies in metropolitan and non-metropolitan areas.

**Timeline**		**Metropolitan area**			**Non-metropolitan area**		
**Year**	**Month**	**Elementary**	**Middle**	**High**	**Elementary**	**Middle**	**High**
2020	3	Closed	Closed	Closed	Closed	Closed	Closed
	5	Partial	Partial	Partial	Partial	Partial	Partial
	8	Closed	Closed	Partial	Partial(1/3)	Partial(1/3)	Partial(1/3)
	9	Partial(1/3)	Partial(1/3)	Partial(2/3)	Partial(1/2)	Partial(1/2)	Partial
	10	Partial(2/3)	Partial(2/3)	Partial	Full	Full	Full
	11	Partial(2/3)	Partial(2/3)	Partial (2/3)	Full	Full	Full
	12	Closed	Closed	Closed	Closed	Closed	Closed
2021	3	Partial (2/3)	Partial(2/3)	Partial(2/3)	Partial(2/3)	Partial(2/3)	Partial(2/3)
	7	Closed	Closed	Closed	Partial	Partial	Partial
	8	Partial	Partial	Partial	Full	Full	Full
	11	Full	Full	Full	Full	Full	Full
	12	Partial(5/6)	Partial(5/6)	Partial(2/3)	Full	Full	Full

In March 2020, in response to the rapid spread of COVID-19, the South Korean government temporarily closed all schools nationwide and shifted to complete learning. As the situation improved, the government began partially reopening schools in May 2020, based on regional infection rates. High school seniors were prioritized for in-person classes to prepare for college entrance exams. At the same time, other grades attended on a rotating schedule, either weekly or every other day, using a hybrid model of in-person and remote learning. The school closure policies for metropolitan and non-metropolitan areas are summarized in Table 5.

**Table 5:** Selected region-specific superspreading events in South Korea with reported date of index case.

**Region**	**Cluster**	**Cluster size**	**Reported date of Index case**	**References**
Seoul	Church	563	3 August 2020	(47)
Seoul	Rally	226	19 August 2020	(48)
Seoul	Jail	1,052	28 November 2020	(49)
Seoul	Restaurant	263	29 November 2020	(50)
Seoul	University Hospital	268	12 February 2021	(51)
Incheon	Distribution center	181	8 May 2020	(45)
Daegu	Church	832	18 February 2020	(40)
Daegu	Private meeting, Bar	415	10 May 2021	(52)
Ulsan	Convalescent facility	189	5 December 2020	(53)
Gwangju	Convalescent facility	175	1 January 2021	(54)
Gwangju	Church	212	14 December 2021	(55)
Gwangju	Church	149	24 January 2021	(56)
Daejeon	Mission school	157	20 January 2021	(57)

## 3 Results

In this section, we constructed age-specific and region-specific networks, enabling the direct calculation of the empirical effective reproduction number from the resulting infection tree. This approach highlights the importance of empirical estimates of $R_{t}$, providing a more accurate reflection of transmission dynamics across different demographics and regions.

### 3.1 Age-specific empirical $R_{t}$

**Figure 4:**
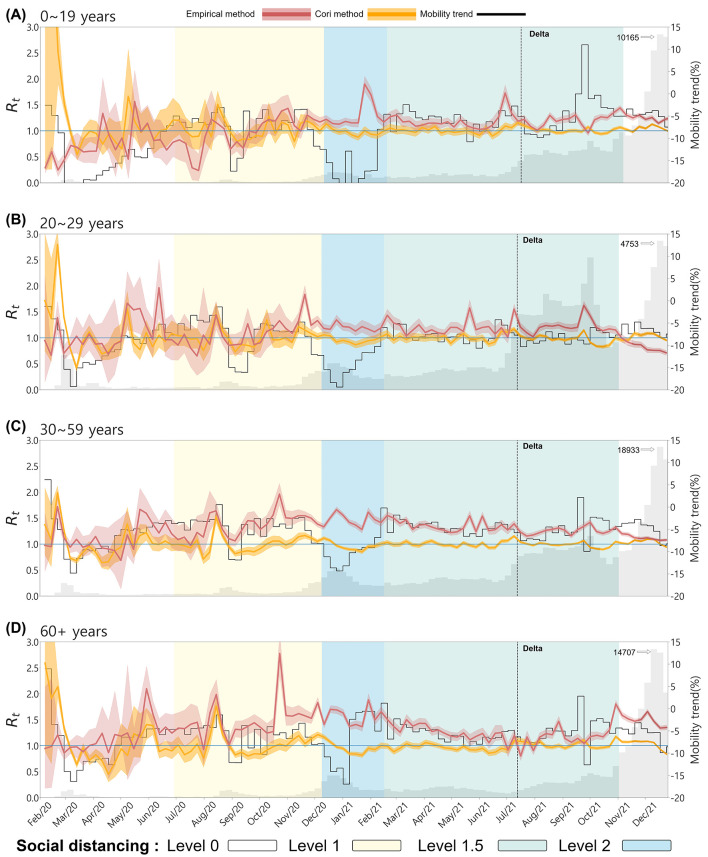
The effective reproduction number and mobility trends by Age. **(A)** 0–19, **(B)** 20–29, **(C)** 30–59, and **(D)** 60+. The red curve represents the empirical $R_{t}$, the orange curve represents Cori's $R_{t}$. The gray bars indicate the number of confirmed cases and the black curve represents the mobility trend. The background color of the graph represents the social distancing levels based on the non-metropolitan criterion. The confidence interval of both are set between the 5th and 95th percentiles.

Understanding the temporal variations in the effective reproduction number across different age groups provides crucial insights into age-specific transmission dynamics and the impact of social behaviors on disease spread. Figure 4 displays the temporal changes in the effective reproduction number, mobility, and confirmed cases across four age groups. The red curve represents the effective reproduction number empirically calculated from the infection network(Empirical $R_{t}$). In contrast, the orange curve represents the $R_{t}$ estimated using the EpiEstim R package, also known as Cori's method (hereafter referred to as Cori's $R_{t}$). The gray bars indicate the number of confirmed cases and the black curve represents the mobility trend. The black dashed vertical line marks the point at which the Delta variant became dominant, and the background colors of the graph represent the social distancing levels based on the non-metropolitan criterion. The social distancing levels differ between metropolitan and non-metropolitan areas, so the levels in the age-specific graphs are marked according to the non-metropolitan standards. In all age groups, a noticeable difference was observed between Cori's $R_{t}$ and the empirical $R_{t}$ values.

In Figure 4A for the 0–19 age group, despite very few confirmed cases at the onset of the outbreak, Cori's $R_{t}$ is overestimated, with values greater than 1, whereas the empirical $R_{t}$ remains below 1. Both $R_{t}$ values spiked above 1 in the other age groups, indicating the disease's rapid spread at the onset of the outbreak. Mobility trends in the 0–19 age group followed the patterns of school closures and reopening policies (see Table 4). For instance, mobility decreased during school closures, and the both $R_{t}$ values remained relatively stable. After August 2021, when school closure policies were relaxed, mobility increased, particularly in metropolitan areas, but the empirical $R_{t}$ did not rise, indicating that increased movement among younger populations did not lead to an immediate spike in transmission, possibly due to vaccination coverage or reduced susceptibility in certain cohorts.

For the 20–29 and 30–59 age groups, both Cori's $R_{t}$ and empirical $R_{t}$ exhibited similar trends early in the outbreak. However, the empirical $R_{t}$ showed more variability in response to changes in confirmed cases. As the outbreak progressed, particularly during the dominance of the Delta variant, the empirical $R_{t}$ remained consistently above 1 except 20–29 age group, reflecting the continued spread of the virus. At the same time, Cori's $R_{t}$ tended to stabilize around 1. At the onset of the outbreak, mobility in these age groups dropped sharply due to public health interventions and social distancing measures. This decrease in mobility coincided with a sharp increase in both the empirical $R_{t}$ and Cori's $R_{t}$ values, reflecting the initial rapid spread of the virus despite reduced movement. During the period of Delta variant dominance, mobility remained relatively low, while the empirical $R_{t}$ stayed above 1 for 30–59 age group, indicating that even with restricted movement, the transmission of the variant sustained high transmission in this age group. In the 60+ age group, the empirical $R_{t}$ also displayed larger fluctuations than Cori's $R_{t}$, especially from November 2020 to December 2021, when Cori's method showed relatively stable values close to 1. Detailed numerical trends of the empirical and Cori's $R_{t}$ across age groups are summarized in the Appendix Table A2.

This suggests that the empirical method better captured the real-time transmission dynamics and spikes in confirmed cases in older populations, whereas Cori's method smoothed over these fluctuations. For this age group, mobility increased during specific periods, such as January and February 2021, while the empirical $R_{t}$ remained above 1. This indicates that older populations were more mobile at certain times despite restrictions, potentially contributing to sustained transmission.

### 3.2 Region-specific empirical $R_{t}$

Examining the spatial variations in the effective reproduction number ($R_{t}$) provides valuable insights into how transmission dynamics differ across regions. Factors such as population density, mobility patterns, and the effectiveness of contact tracing can significantly influence regional differences in $R_{t}$. By analyzing these variations, we can better understand how localized outbreaks unfold and how public health interventions have shaped the spread of the virus in different geographic areas.

**Figure 5:**
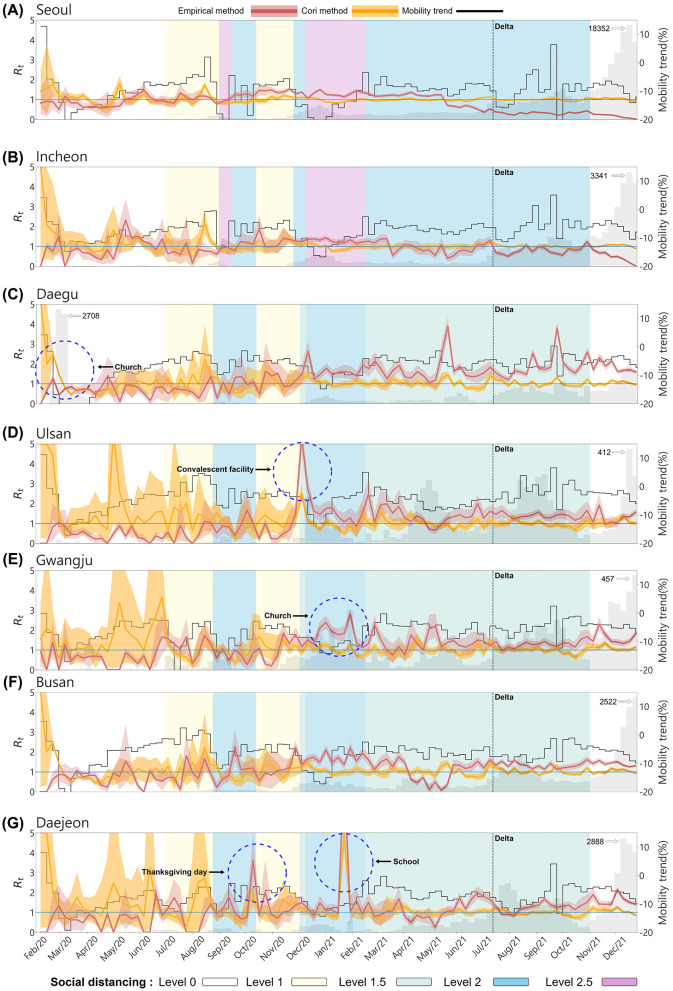
The effective reproduction number and mobility trend by Region. **(A)** Seoul, **(B)** Incheon, **(C)** Daegu, **(D)** Ulsan, **(E)** Gwangju, **(F)** Busan, and **(G)** Daejeon. The red curve represents the effective reproduction number empirically calculated from the infection network, while the orange curve shows $R_{t}$ estimated using the EpiEstim R package. The gray bars indicate the number of confirmed cases and the black curve represents the mobility trend. The background color of the graph represents the social distancing levels based on the non-metropolitan criteria. The metropolitan area is marked as the same for both the enhanced social distancing level 2 and level 2.5. The confidence interval of both are set between the 5th and 95th percentiles.

Figure 5 illustrates the temporal changes in the effective reproduction number, mobility, and confirmed cases across seven regions. The red curve represents Empirical $R_{t}$, while the orange curve represents Cori's $R_{t}$. The gray bars indicate the number of confirmed cases by region, and the black curve shows the mobility trend. The black dashed vertical line marks the point at which the Delta variant became dominant, and the five background colors of the graph represent the social distancing levels. In South Korea, the level of social distancing were applied differently in metropolitan and non-metropolitan areas depending on the spread of COVID-19.

From the onset of the outbreak until the Delta variant became dominant, the empirical $R_{t}$ values were more responsive to regional outbreaks and fluctuations in confirmed cases, accurately reflecting dynamic transmission patterns across all regions. In contrast, Cori's $R_{t}$ displayed a smoother, more stable curve, making it less sensitive to sudden spikes or declines in case numbers. As a result, Cori's method often underestimated transmission peaks, with $R_{t}$ tending to stabilize around 1. Due to very few confirmed cases during the early stages of the outbreak, Cori's method overestimated, $R_{t}$ values, exceeding 1 in most regions. Except for Daegu, Cori's $R_{t}$ values in other regions were overestimated, while empirical $R_{t}$ values remained below 1 until the number of confirmed cases began to rise. Daegu, the epicenter of the early outbreak due to the superspreading event at the Shincheonji Church in February 2020 it exhibited a distinct pattern. During this period, empirical $R_{t}$ accurately predicted the surge in confirmed cases, while Cori's $R_{t}$ showed and initial overestimation that gradually decreased.

Superspreading events occurred not only in the Daegu region but across the entire country (see Table 5). The empirical method more effectively captured real-time transmission dynamics and spikes in confirmed cases, such as during superspreading events. In Ulsan, during the social distancing Level 1.5 period around December 2020, mobility decreased substantially, yet empirical $R_{t}$ soared to 5, reflecting a massive outbreak at a convalescent facility. Gwangju displayed unique dynamics, with empirical $R_{t}$ remaining zero between May and July 2020, indicating very low transmission. However, in January 2021, when an SSE occurred at a church, the empirical $R_{t}$ value correctly predicted the increase in cases. Moreover, Daejeon experienced two major superspreading events: one during Thanksgiving (October 2020) and another at a school (between January 2021 and February 2021). In both instances, empirical $R_{t}$ rose sharply before confirmed cases surged.

After the Delta variant became dominant, constructing infection networks for metropolitan areas posed challenges due to the large urban population sizes. The scale of the cities made effective contact tracing more difficult, likely affecting the accuracy of empirical $R_{t}$ values in these regions. As seen in Figures 5A, B, in metropolitan areas like Seoul and Incheon, despite the rapid increase in confirmed cases, the empirical $R_{t}$ decreases to below 1. This reflects the challenges of accurately tracing transmission routes in densely populated regions. The number of confirmed cases and rapid transmission overwhelmed the contact tracing efforts in these cities, leading to potential underreporting or incomplete data on infection links. This could result in lower or delayed empirical $R_{t}$ values, as the full scope of transmission events may not have been captured in real time. Detailed regional trends of the empirical and Cori's $R_{t}$ estimates are provided in Appendix Tables A3, A4.

However, non-metropolitan areas with smaller populations were more likely to maintain accurate contact tracing, resulting in more consistent and reliable $R_{t}$ values. The difference in tracing effectiveness between metropolitan and non-metropolitan regions likely contributed to the observed discrepancies in infection dynamics across these regions, particularly during the dominance of the Delta variant's. Thus, the large-scale population on contact tracing in metropolitan areas likely impacted the accuracy of the infection network and, consequently, the calculated $R_{t}$ values in those regions. Interestingly, there was no clear correlation between mobility trends and $R_{t}$. Specifically, only in Ulsan was a temporary spike in empirical $R_{t}$ during holidays, such as the Lunar New Year, when mobility increased. This signifies that factors beyond mobility, such as public health interventions and the effectiveness of contact tracing, played a more critical role in transmission control.

To complement our earlier comparisons between our proposed empirical approach and Cori's method, we conducted additional analyses using the Wallinga-Teunis (WT) method (34). As illustrated in Appendix Figures A2, A3, our method more effectively captures local and temporal variations in transmission dynamics—particularly during superspreading events and periods of low incidence—compared to the WT method. By contrast, the WT method often fails to produce estimates in low-incidence settings, as observed in Daejeon during the initial stages of the outbreak. These results underscore the robustness and practical utility of our empirical $R_{t}$ estimation framework.

Furthermore, we performed additional validation to demonstrate that our method remains robust in the presence of incomplete data—a common issue arising from untraceable cases, such as pre-symptomatic or asymptomatic infections in COVID-19. Specifically, we ran simulations using an Agent-Based Model (ABM) on a random synthetic network of 10,000 individuals with a fixed degree, 4. Through an SIR framework, we generated an infection network that enabled us to compute the empirical effective reproduction number ($R_{t}$) and compare it with both the theoretical basic reproduction number ($R_{0}$) (58) and Cori's $R_{t}$ (Appendix Figure A1, Table A1). Our results indicate that our empirical approach provides more accurate estimates of the effective reproduction number than Cori's $R_{t}$ during the early stages of an epidemic, while remaining consistent with the theoretical basic reproduction number (1.5) under varying levels of data completeness. Moreover, we observed that the presence of incomplete data did not significantly compromise the accuracy of our method within this random network setup. Although we have so far examined only random networks, different network structures may influence empirical $R_{t}$ estimates, and we intend to explore these variations in future research.

## 4 Discussion

Accurate estimation of the effective reproduction number ($R_{t}$) is crucial for guiding timely and impactful public health interventions during epidemics such as COVID-19 (35, 36). In this study, we introduce an innovative method for estimating the empirical $R_{t}$ by constructing infection networks from detailed transmission data. This network-based approach represents a powerful alternative to traditional methods—such as Cori's $R_{t}$ and Bayesian filtering techniques—which typically assume homogeneous transmission across a population. In filtering-based methods, compartmental models (e.g., SIR) are combined with statistical filtering and inherently assume uniform mixing within the population. Such assumptions can introduce significant inaccuracies, particularly in the context of COVID-19, where transmission dynamics differ widely across age groups and regions, and vary in response to public health interventions (37).

By directly incorporating the inherent variability in transmission, our infection network-based methodology addresses these challenges more effectively than existing models. Compared to established network-based approaches (34) or structured population models (38, 39), our method offers practical advantages. For example, the Wallinga–Teunis (WT) approach constructs probabilistic infection trees based only on aggregated case counts and serial interval distributions, whereas our approach uses empirical contact patterns and temporal information to reconstruct the actual transmission network. This grounding in observed data captures real-world dynamics more precisely. Similarly, structured-population models stratify individuals into subgroups based on select features, estimating within-group and between-group transmission. Our method, in contrast, operates at the individual level, incorporating actual contact data to build an empirical infection network. This granularity enables a more accurate portrayal of transmission pathways.

Leveraging extensive transmission records from the Korea Disease Control and Prevention Agency (KDCA) during the first two years of the pandemic, we found that the empirical $R_{t}$ exhibited sharper fluctuations than Cori's $R_{t}$, thus reflecting sudden spikes in confirmed cases with higher fidelity. In contrast, Cori's estimates were smoother and less responsive to abrupt changes, often underrepresenting transmission peaks—particularly during short-term surges or explosive outbreaks. This divergence was especially notable in the early outbreak in Daegu, where a superspreading event at the Shincheonji Church triggered a rapid rise in transmission (40). While our empirical $R_{t}$ surged in tandem with the outbreak, Cori's $R_{t}$ initially overestimated overall transmission and then declined slowly, overlooking the rapid escalation observed on the ground. Furthermore, during the initial stages of the epidemic—when case numbers remained low—Cori's $R_{t}$ often exceeded 1, whereas the empirical $R_{t}$ consistently stayed below 1 until case counts began to climb, aligning more closely with real-world transmission patterns.

Our network-based approach also demonstrates a distinct capacity to evaluate non-pharmaceutical interventions (NPIs), which may be obscured by temporal smoothing in traditional incidence-based methods. Regions that implemented stringent social distancing and quarantine measures experienced $R_{t}$ values dropping below 1 within just a few weeks, in stark contrast to regions with looser restrictions, where $R_{t}$ remained above 1 for longer periods. These findings underscore the utility of timely and highly resolved $R_{t}$ monitoring, particularly in settings characterized by substantial regional variability. This granularity allows public health authorities to make faster, more informed decisions about when to intensify or relax NPIs in order to contain outbreaks effectively. Another major advantage of our empirical approach is the ability to pinpoint superspreading events, thereby illuminating the specific transmission pathways that fuel rapid case escalation. Identifying high-risk individuals and locations enables more targeted and resource-efficient intervention strategies. The reconstruction of infection networks, therefore, not only refines real-time $R_{t}$ estimates but also offers critical insights into preventing further spread in vulnerable communities.

Despite these strengths, our method is constrained by the limitations of available contact tracing data. During high caseload periods in metropolitan areas (e.g., Seoul and Incheon), contact tracing systems were frequently overwhelmed, leaving numerous confirmed cases without reliable infector–infectee linkages. This incomplete dataset can bias empirical $R_{t}$ estimates downward if fewer secondary infections are observed. Additionally, partially connected or disconnected (singleton) nodes may distort the perceived network structure, particularly during large outbreaks or underreporting of asymptomatic cases. Consequently, while our approach delivers fine-grained insights when contact data are robust, its interpretability must be contextualized according to tracing efficacy and reporting quality. Moreover, heterogeneous transmission drivers—such as individual behavior, population density, and viral variants—can produce patterns not fully captured in our networks (41–44).

Recognizing these limitations, future research should concentrate on enhancing data completeness and refining network construction methods. Incorporating more robust data inputs, such as improved contact tracing, testing protocols, and real-time mobility patterns, could reduce data gaps and enable even more precise $R_{t}$ estimates. Nevertheless, our findings highlight the importance of integrating detailed infection networks into epidemic modeling to obtain more accurate, context-specific insights into disease transmission. By doing so, public health decision-makers gain a stronger basis for intervention planning, tailored to the dynamic and heterogeneous nature of epidemics. Overall, our infection network-based method for estimating $R_{t}$ represents a critical advancement in epidemiological analysis. By eschewing the homogeneous-mixing assumptions of traditional models and leveraging rich, individual-level data, we offer a tool that can capture epidemic dynamics more sensitively and accurately. This improved estimation of $R_{t}$ is vital for forecasting outbreak trends, assessing the impact of NPIs, and guiding strategic allocations of public health resources—particularly in rapidly evolving epidemic settings.

## Data Availability

The data analyzed in this study is subject to the following licenses/restrictions: the data used in the current study were obtained from the Korea Disease Control and Prevention Agency (KDCA) and are not publicly available. Requests to access these datasets should be directed to sunmilee@khu.ac.kr.

## Footnotes

^1^WHO (2025). Available online at: https://covid19.who.int/ (accessed March 3, 2025).

^2^KDCA (2023). Available online at: https://dportal.kdca.go.kr/pot/cv/trend/dmstc/selectMntrgSttus.do (accessed March 3, 2025).
