# Supplementary material for: A novel approach to estimating ℛt through infection networks: understanding regional transmission dynamics of COVID-19
Source: Front Public Health. 2025 Jun 18;13:1586786. doi: 10.3389/fpubh.2025.1586786 (PMC12213828; doi:10.3389/fpubh.2025.1586786)
Supplement: Supplementary file 1 [file Supplementary_file_1.pdf]

APPENDIX

In this Appendix, we begin by describing the algorithm used to estimate the empirical reproduction number $\mathcal{R}_t$. Next, we present the results of our Agent-Based Model (ABM) for estimating the empirical $\mathcal{R}_t$ under a synthetic network structure, including scenarios with incomplete data. We then compare our empirical $\mathcal{R}_t$ estimates to those obtained using the Wallinga–Teunis (WT) method. Finally, we provide numerical summaries corresponding to the trends shown in Figures 4 and 5.

First, we outline the steps for estimating the empirical reproduction number $\mathcal{R}_t$ in the infection network, as described in the Methods section.

Algorithm 1 Estimating the empirical reproduction number in the infection network

Require: $G = (V, E)$

$E_{count} \leftarrow |E|$
$V_{count} \leftarrow |V|$
$S_{count} \leftarrow 0$
for each node v in V **do**
    if out degree(v) = 0 **then**
        $S_{count} \leftarrow S_{count} + 1$
    end if
end for
if $N_{count} - S_{count} \neq 0$ **then**
    $\mathcal{R}_t \leftarrow E_{count}/(N_{count} - S_{count})$
end if

Next, we present the results of our Agent-Based Model (ABM) for estimating the empirical $\mathcal{R}_t$ under a synthetic network structure, including scenarios with incomplete data. In the context of a random regular network, the theoretical basic reproduction number $\mathcal{R}_0$ is defined as:

$$\mathcal{R}_0 = \frac{\beta}{\gamma + \beta} \cdot (k - 1), \tag{1}$$

where β is the transmission rate, γ is the recovery rate, and k denotes the degree of each node. In our simulation study, both β and γ were set to 0.125, and each node was assigned a fixed degree of $k = 4$. The total population size was 10,000 nodes, and the simulation was repeated 100 times. Under these parameters, the synthetic network has a theoretical basic reproduction number of 1.5. We also explored scenarios ranging from fully connected infection networks—representing an idealized setting—to partially disconnected structures, which model conditions before and after the emergence of the Delta variant. As illustrated in Appendix Figure 1, our empirical approach closely matches the theoretical expectations and maintains robust, consistent estimates even when the infection network data are incomplete. During the early phase of an epidemic, it provides more accurate reproduction number estimates than Cori's method. However, because real-world contact networks often deviate from the structure of random regular networks, such differences may reduce the accuracy of our empirical $\mathcal{R}_t$ estimates. Future research will focus on refining and validating this approach under more realistic network conditions.

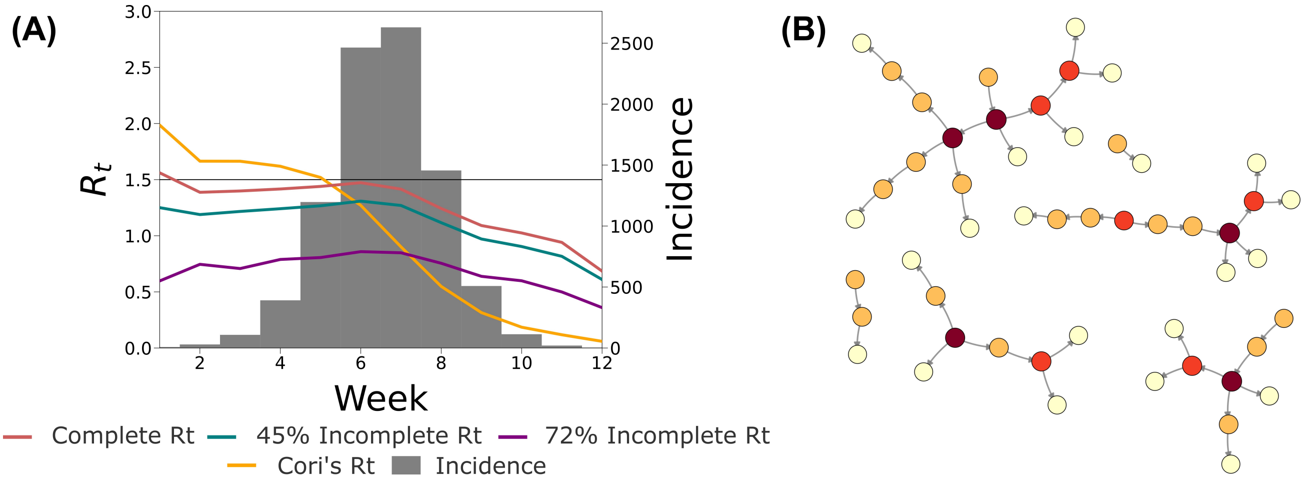

Figure 1. **(A)** We compare our empirical $\mathcal{R}_t$ estimates—derived under three levels of data completeness: complete (red), 45% incomplete (green), and 72% incomplete (purple)—to those obtained using Cori's method (orange). The theoretical $\mathcal{R}_0$ is shown as a black line. **(B)** We illustrate the resulting infection networks used to estimate the empirical effective reproduction number, including a fully observed network. Node color intensity indicates node degree, with darker hues representing higher degrees. Two incomplete networks (with 45% and 72% missing data, respectively) were randomly selected from the full network.

Table 1. We compare the confidence intervals of the empirical $\mathcal{R}_t$ estimates and Cori's $\mathcal{R}_t$ estimates for the random network described above.

Complete data		45% incomplete data		72% incomplete data		Cori's $\mathcal{R}_t$	
CI 5%	CI 95%	CI 5%	CI 95%	CI 5%	CI 95%	CI 5%	CI 95%
0.60	2.17	0.49	1.89	0.02	1.47	0.72	2.61
0.61	2.19	0.51	1.92	0.10	1.32	0.73	2.59
0.64	2.19	0.56	1.92	0.30	1.28	0.73	2.50
0.66	2.22	0.58	1.96	0.35	1.26	0.67	2.36
0.68	2.27	0.60	2.02	0.38	1.33	0.47	2.07
0.64	2.19	0.57	1.97	0.38	1.32	0.17	1.63
0.52	1.96	0.47	1.77	0.31	1.20	0.00	1.11
0.46	1.72	0.41	1.54	0.24	1.03	0.00	0.66
0.46	1.59	0.40	1.41	0.23	0.97	0.00	0.38
0.23	1.65	0.19	1.44	0.00	1.12	0.00	0.28
0.00	1.70	0.00	1.52	0.00	1.19	0.00	0.20
0.00	1.12	0.00	1.02	0.00	0.67	0.00	0.14

Lastly, next two figures compare our empirical $\mathcal{R}_t$ estimates to those obtained using the Wallinga–Teunis (WT) method. Furthermore, we provide three tables of numerical summaries corresponding to the trends shown in Figures 4 and 5.

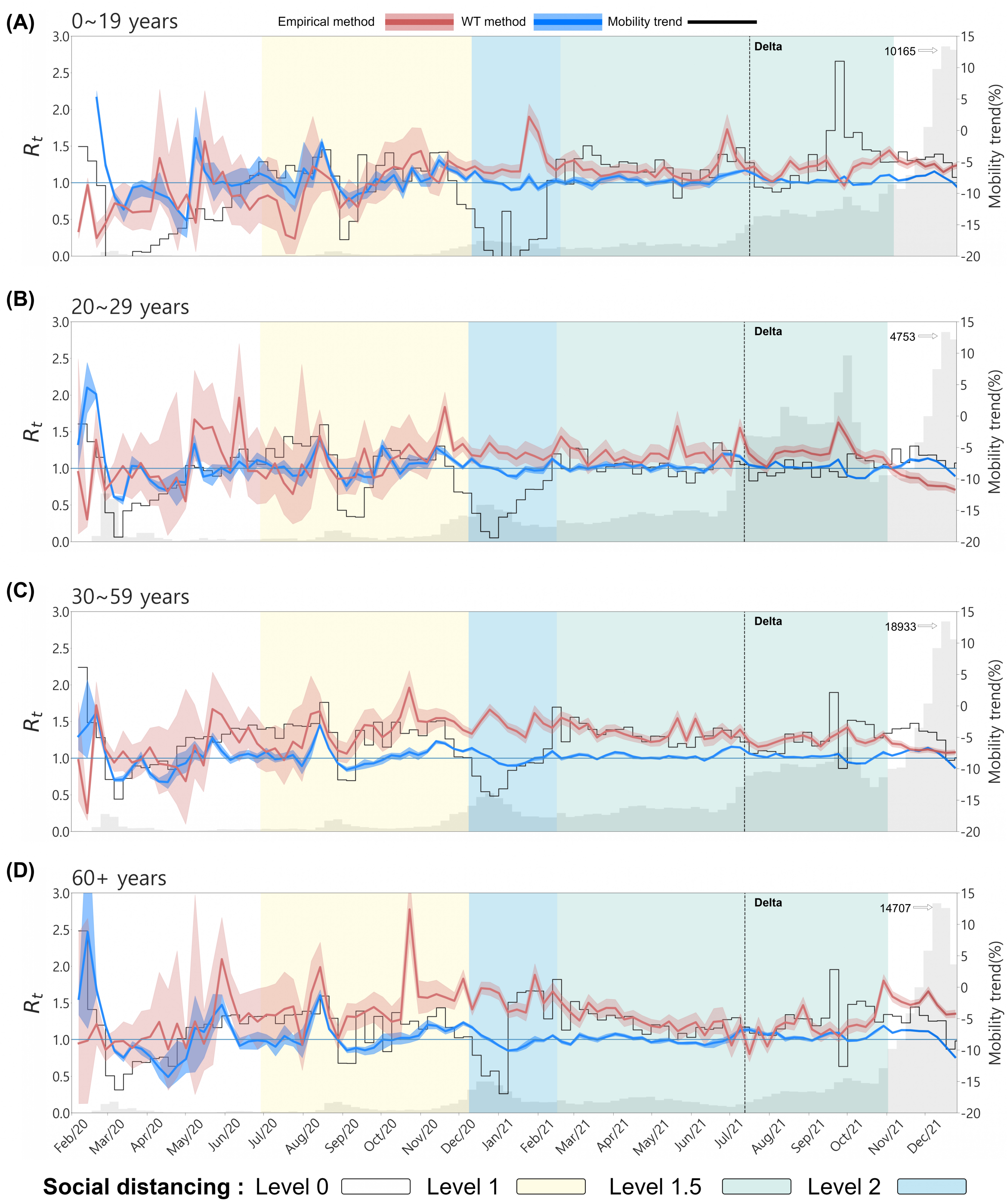

Figure 2. Comparison of the empirical $\mathcal{R}_t$ (red) and Wallinga–Teunis (WT) method $\mathcal{R}_t$(blue) across four age groups. Gray bars represent the number of confirmed cases, and the black curve indicates weekly mobility trends. Background colors show the levels of social distancing based on non-metropolitan criteria. The empirical method demonstrates greater sensitivity to real-time transmission fluctuations, particularly during superspreading events and periods of low incidence, where the WT method often fails to generate estimates. The confidence interval of both are set between the 2.5% and 97.5%.

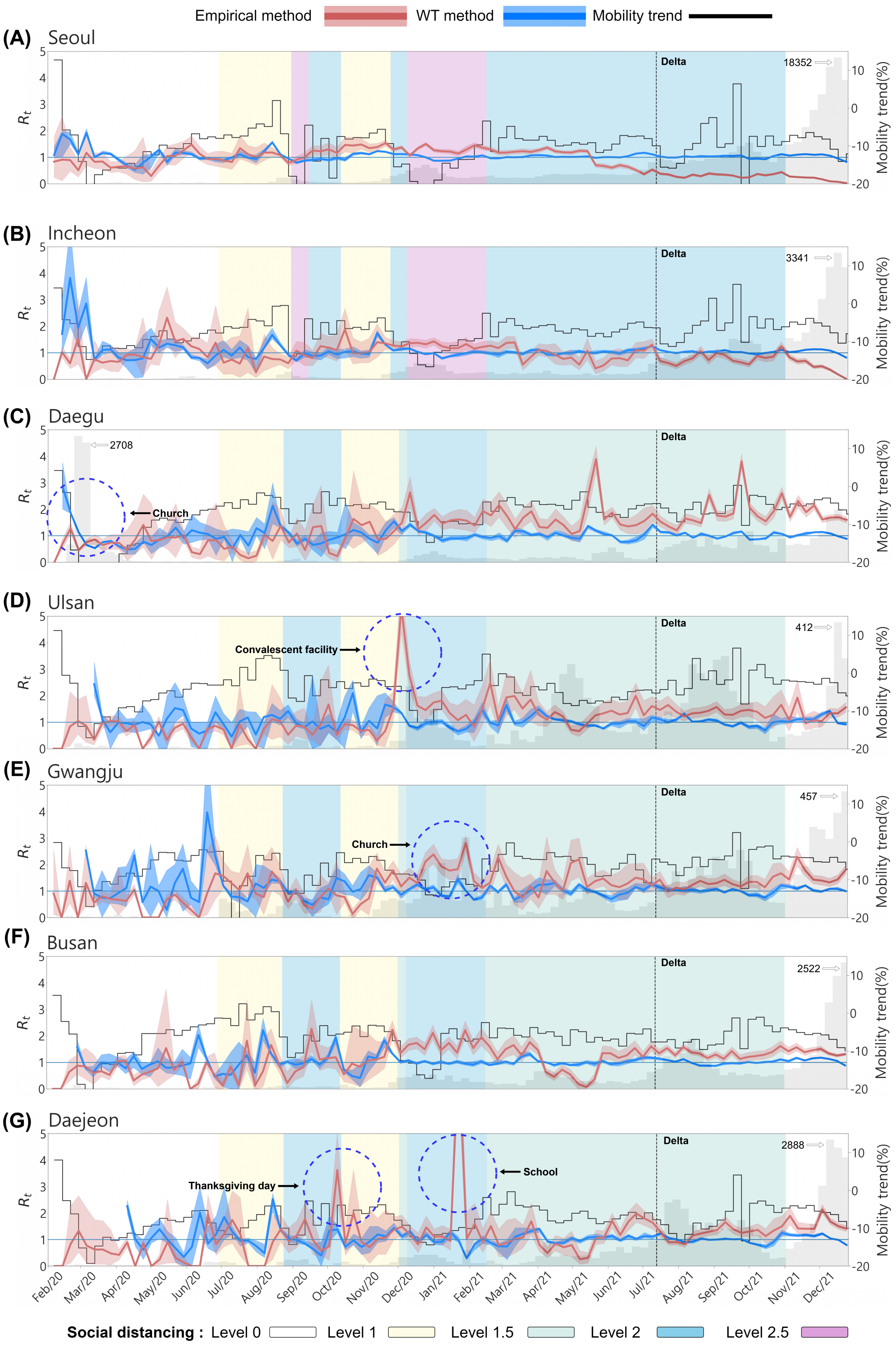

Figure 3. Comparison of the empirical $\mathcal{R}_t$ (red) and Wallinga–Teunis (WT) method $\mathcal{R}_t$ (blue) across seven regions. Gray bars represent the number of confirmed cases, and the black curve indicates weekly mobility trends. Background shading denotes regional social distancing levels. The empirical method more effectively captures sudden changes in transmission, whereas the WT method tends to produce gaps in estimates under sparse data conditions, highlighting the empirical method's robustness in regional analyses. The confidence interval of both are set between the 2.5% and 97.5%.

Table 2. Weekly trends of the empirical $\mathcal{R}_t$ and Cori's $\mathcal{R}_t$ across four age groups. Each value represents the reproduction number estimated for a 7-day window. This table highlights discrepancies between the empirical and Cori's methods, particularly during early outbreak phases and periods of low incidence.

	Empirical Rt				Cori's Rt			
Week	0-19	20-29	30-59	60+	0-19	20-29	30-59	60+
1	0.29	0.95	0.97	0.95	5.00	1.72	1.37	2.59
2	0.59	0.66	0.95	0.99	5.00	1.38	1.09	1.91
3	0.24	1.39	1.72	1.21	2.51	2.80	1.99	2.14
4	0.45	0.70	1.13	0.86	1.51	1.41	1.28	1.33
5	0.72	0.85	0.94	0.97	0.96	0.79	0.73	0.94
6	0.68	1.03	1.09	0.98	0.54	0.42	0.68	0.64
7	0.59	0.87	0.94	0.88	0.85	0.92	0.84	0.96
8	0.61	1.07	0.98	1.00	0.99	1.11	0.96	0.72
9	0.61	0.88	1.15	1.04	0.95	0.90	0.84	0.83
10	1.34	0.89	0.92	1.24	0.74	0.69	0.64	0.71
11	0.91	0.68	0.91	0.86	0.95	0.74	0.66	0.45
12	0.63	0.87	0.87	1.22	0.78	0.88	0.87	0.67
13	0.86	0.93	0.68	1.18	0.65	0.94	0.94	0.94
14	0.45	1.67	1.18	1.25	1.66	1.55	1.39	0.81
15	1.56	1.54	0.90	0.94	1.18	0.90	0.94	1.23
16	0.98	1.56	1.67	1.25	0.97	0.77	1.16	1.16
17	1.15	1.23	1.59	2.10	1.12	1.10	1.22	1.41
18	0.81	0.97	1.39	1.61	0.83	0.83	1.01	1.39
19	0.83	1.96	1.21	1.24	1.00	1.05	0.99	0.89
20	0.63	0.96	1.42	1.36	1.03	0.93	1.06	0.94
21	0.78	0.96	1.20	1.24	1.21	1.08	0.99	0.90
22	0.82	0.86	1.08	1.34	1.20	1.04	0.99	1.02
23	0.76	1.07	1.13	1.33	0.99	1.03	0.97	0.83
24	0.29	0.80	0.97	1.42	0.89	0.97	0.92	0.89
25	0.24	0.65	1.22	1.45	0.89	0.99	1.08	1.04
26	0.88	1.05	1.13	0.93	1.16	0.76	0.71	0.76
27	1.22	0.88	1.60	1.63	0.88	1.01	0.84	1.18
28	1.14	1.45	1.64	1.99	1.51	1.46	1.52	1.77
29	1.04	1.02	1.28	1.33	1.21	1.13	1.17	1.21
30	0.65	0.85	1.10	1.01	0.97	0.97	0.95	1.01
31	0.79	0.87	1.06	1.32	0.80	0.73	0.81	0.82
32	0.66	0.88	1.25	1.31	0.84	0.87	0.85	0.87
33	0.99	1.27	1.45	1.38	0.83	0.85	0.87	0.80
34	0.96	0.87	1.44	1.44	0.91	0.82	0.85	0.85
35	1.15	0.91	1.28	1.35	0.89	0.96	0.91	0.84
36	1.22	1.12	1.38	1.24	1.28	1.35	1.00	0.91
37	1.19	1.18	1.56	1.27	0.95	0.96	1.06	0.98
38	1.38	1.33	1.96	2.78	1.17	0.96	1.06	1.03
39	1.43	1.18	1.50	1.58	1.11	1.00	0.98	0.99
40	1.10	1.08	1.49	1.57	0.79	0.91	1.03	1.11

Table 3. Weekly trends of the empirical $\mathcal{R}_t$ across seven regions. Each value is computed based on a 7-day moving window using empirical infection network data. The table illustrates spatial heterogeneity in transmission dynamics, with substantial variability across regions.

Week	Seoul	Incheon	Daegu	Ulsan	Gwangju	Busan	Daejeon
1	0.83	0.00	0.00	0.00	0.91	0.00	0.00
2	0.91	1.01	0.70	0.00	0.00	0.00	0.00
3	0.89	0.75	1.27	0.83	1.39	0.63	0.83
4	0.51	1.54	0.46	1.04	0.00	0.87	1.33
5	1.17	0.00	0.76	0.87	1.30	0.85	0.77
6	0.83	0.75	0.85	0.45	0.44	0.61	0.62
7	0.84	0.92	0.64	0.44	0.75	1.07	0.62
8	0.61	0.68	0.74	0.62	0.60	0.75	0.48
9	0.64	0.66	0.72	0.37	0.67	0.84	0.90
10	0.66	0.44	0.45	0.22	0.69	0.83	0.69
11	0.57	0.33	0.86	0.25	0.66	0.33	0.00
12	0.62	0.83	1.41	0.00	0.00	0.23	0.93
13	0.64	0.35	0.80	0.49	0.00	0.94	0.00
14	0.68	1.32	0.12	0.88	0.00	0.77	0.84
15	1.04	2.32	0.94	0.36	0.88	1.54	0.91
16	1.05	1.43	0.93	0.85	0.61	1.07	0.67
17	1.26	1.52	0.80	0.29	0.31	0.32	0.80
18	1.48	1.25	0.50	0.22	0.72	0.00	0.00
19	1.13	1.14	0.15	0.00	0.00	1.06	0.00
20	0.93	0.83	0.82	0.38	0.91	0.63	1.96
21	0.94	1.38	0.83	0.23	1.67	0.53	0.83
22	0.88	0.62	0.50	0.55	1.56	0.73	1.21
23	0.82	0.74	0.78	0.00	1.30	0.71	1.74
24	1.34	0.59	0.70	0.91	1.03	0.13	1.20
25	1.11	1.18	0.69	0.28	1.67	1.82	0.00
26	0.99	0.16	0.56	0.00	0.32	0.97	0.00
27	1.18	0.86	0.26	0.39	0.77	0.67	0.00
28	1.18	0.76	0.80	1.11	1.74	1.66	0.91
29	1.08	0.75	1.37	1.04	1.34	0.92	1.39
30	0.78	0.60	0.28	0.53	0.60	0.22	0.93
31	0.91	1.08	1.08	0.88	1.06	0.66	1.16
32	1.01	0.91	0.45	0.83	0.47	0.82	1.78
33	1.25	0.80	1.32	0.40	1.00	2.27	0.98
34	1.22	1.25	1.36	0.91	0.77	1.11	0.67
35	1.30	1.19	0.96	1.67	0.26	1.16	0.74
36	1.16	1.10	1.09	1.01	0.83	1.19	3.62
37	1.46	1.88	0.48	0.23	1.09	0.54	0.86
38	1.44	0.99	1.63	1.01	0.30	0.95	2.00
39	1.48	0.99	1.40	0.23	0.19	0.83	0.91
40	1.34	1.02	1.52	0.22	0.71	1.07	0.71

Table 4. Weekly trends of Cori's $\mathcal{R}_t$ across seven regions. Each value represents the reproduction number estimated using incidence-based methods with a 7-day window. Comparison with Table 3 underscores the differences in sensitivity and responsiveness between incidence-based and network-based estimation methods.

Week	Seoul	Incheon	Daegu	Ulsan	Gwangju	Busan	Daejeon
1	1.42	5.00	5.00	5.00	1.56	5.00	5.00
2	1.58	2.07	2.00	5.00	2.10	2.00	5.00
3	1.76	1.74	2.48	4.43	1.82	2.48	2.14
4	1.22	1.58	1.44	1.31	0.55	1.44	1.16
5	1.03	0.90	0.79	0.52	1.53	0.79	0.80
6	1.16	1.31	0.45	0.97	0.96	0.45	1.16
7	1.06	1.20	0.73	1.10	1.10	0.73	1.76
8	1.21	1.23	0.70	1.04	1.06	0.70	1.22
9	0.94	0.85	0.71	1.00	1.20	0.71	1.05
10	0.74	0.74	0.46	1.41	0.60	0.46	1.01
11	0.56	0.79	0.45	0.95	1.25	0.45	0.84
12	0.54	1.14	0.79	0.70	0.89	0.79	1.57
13	0.97	1.83	0.66	5.59	1.78	0.66	2.47
14	1.80	1.20	0.99	2.61	3.38	0.99	1.79
15	0.89	1.07	1.05	1.64	2.04	1.05	1.41
16	0.94	1.11	0.80	0.82	1.73	0.80	1.25
17	1.10	1.50	1.16	1.38	1.47	1.16	1.39
18	1.27	1.06	1.03	1.03	0.97	1.03	0.80
19	0.88	1.00	0.93	1.22	2.01	0.93	3.26
20	0.89	0.69	1.18	1.57	2.72	1.18	1.39
21	0.94	0.83	0.88	1.60	3.67	0.88	0.90
22	0.82	1.39	1.28	0.64	1.36	1.28	0.94
23	0.88	0.88	0.45	1.82	0.85	0.45	0.78
24	1.07	0.84	1.23	1.56	0.71	1.23	0.71
25	1.09	0.93	0.76	2.00	0.63	0.76	0.32
26	0.77	0.82	1.48	0.64	0.26	1.48	1.22
27	1.12	1.01	0.96	1.47	0.97	0.96	2.53
28	1.73	2.16	2.23	1.35	1.68	2.23	2.71
29	1.14	1.28	1.35	1.12	1.22	1.35	1.62
30	0.98	1.00	1.31	1.53	1.03	1.31	0.83
31	0.77	0.75	0.62	1.09	1.05	0.62	1.03
32	0.84	0.81	0.90	1.03	0.84	0.90	0.98
33	0.87	0.91	0.54	0.22	0.34	0.54	0.71
34	0.89	0.85	0.56	0.96	0.88	0.56	0.43
35	0.83	0.99	1.11	1.27	0.70	1.11	1.29
36	1.02	0.93	1.02	0.61	1.35	1.02	1.72
37	0.92	1.05	0.78	1.13	1.67	0.78	1.02
38	0.94	0.91	1.43	1.43	1.21	1.43	0.94
39	1.14	1.03	1.35	1.17	1.04	1.35	0.83
40	1.04	0.95	1.09	1.02	0.99	1.09	0.90
